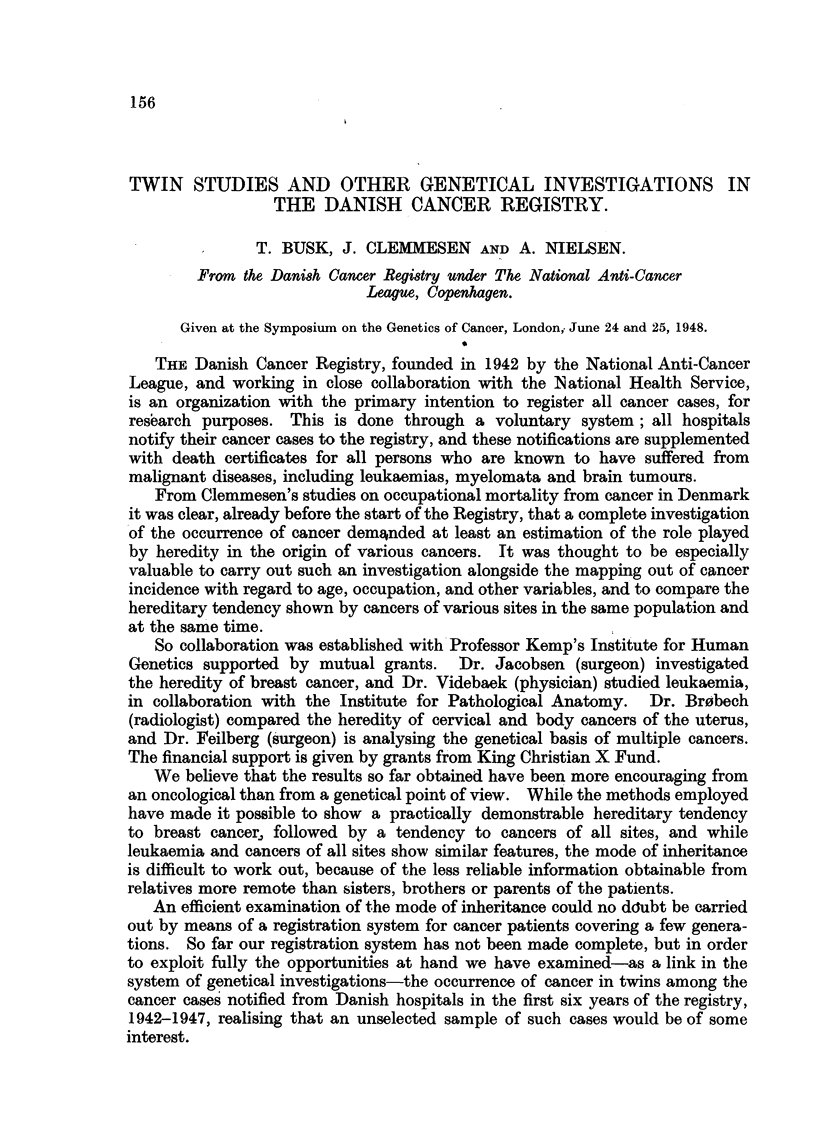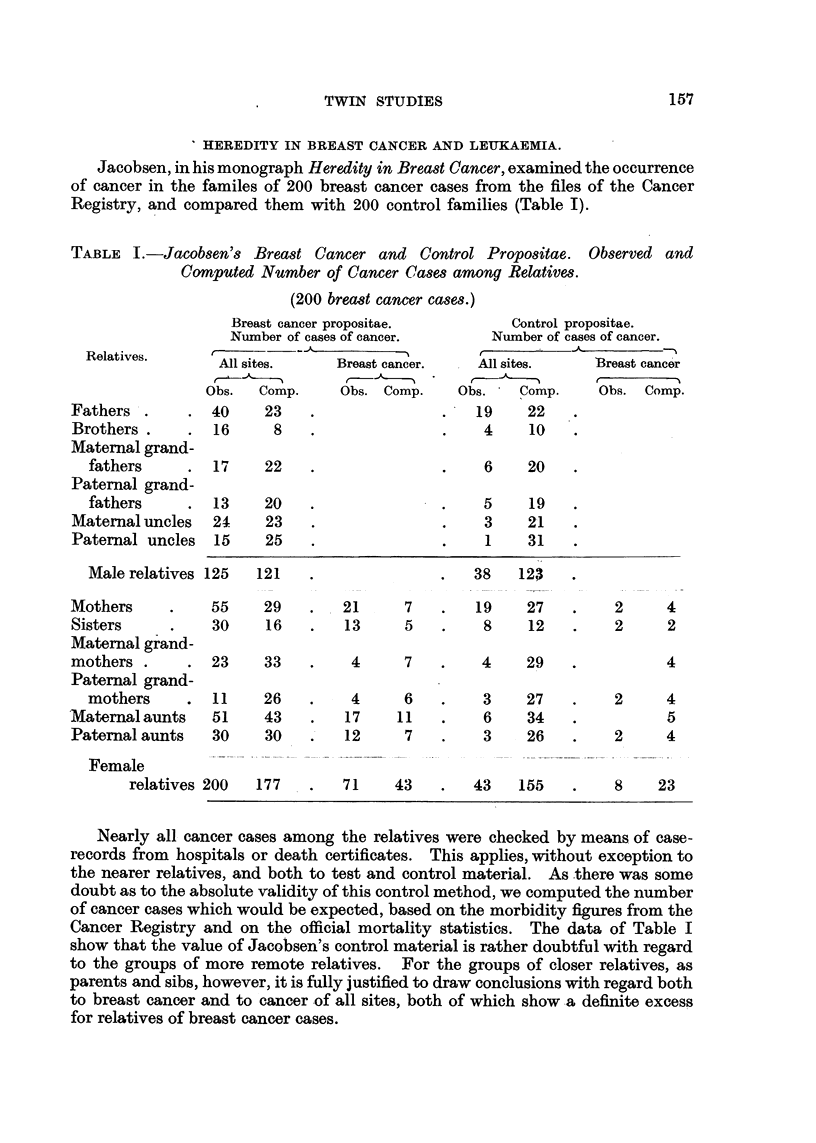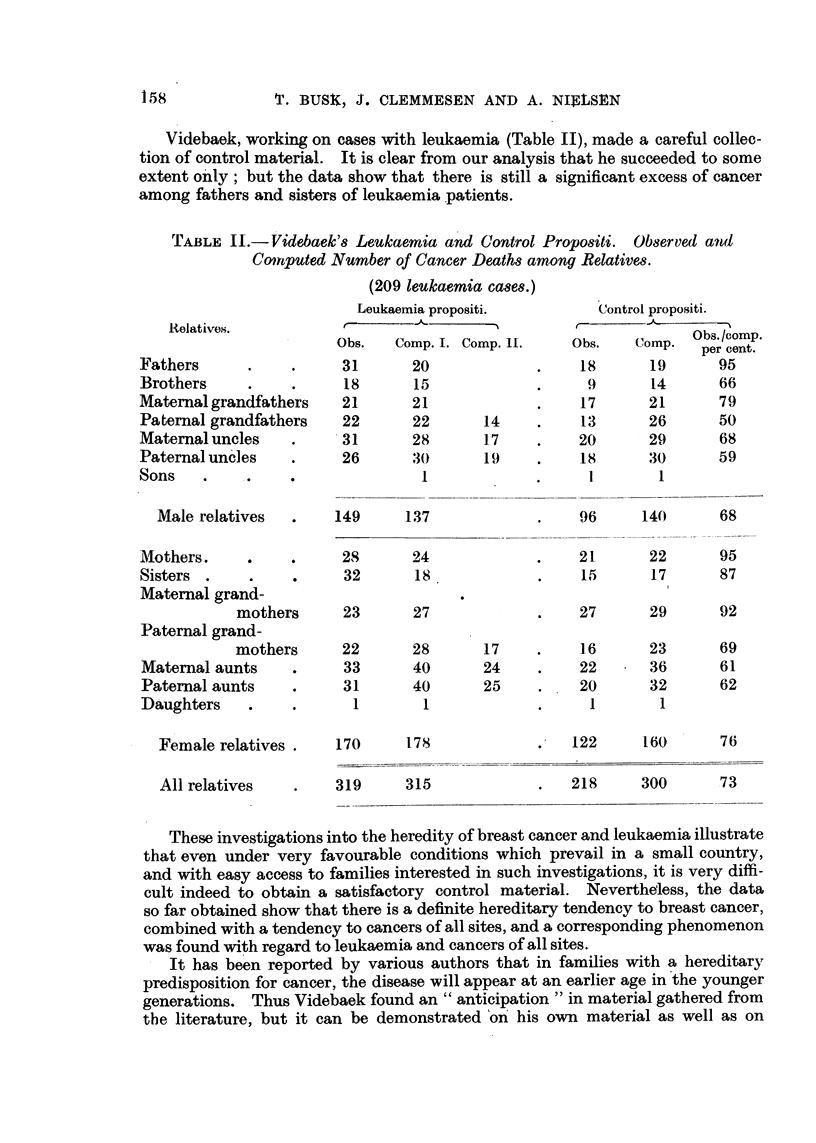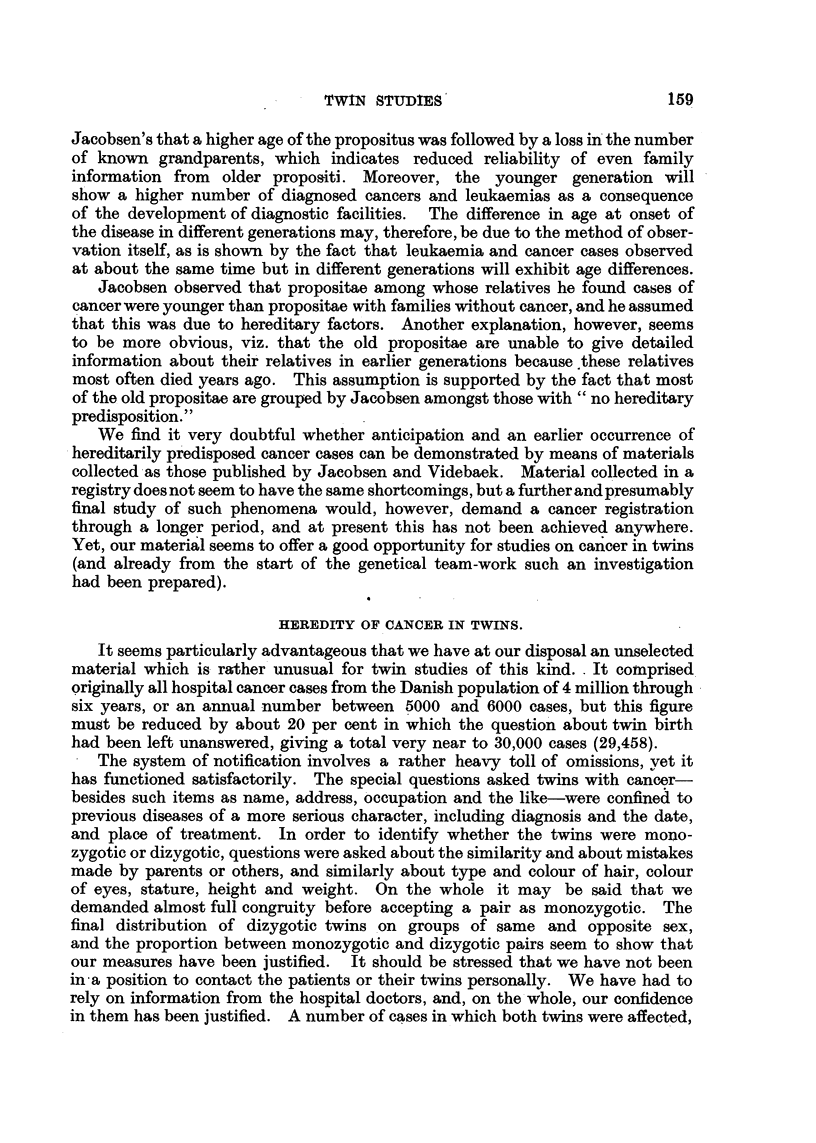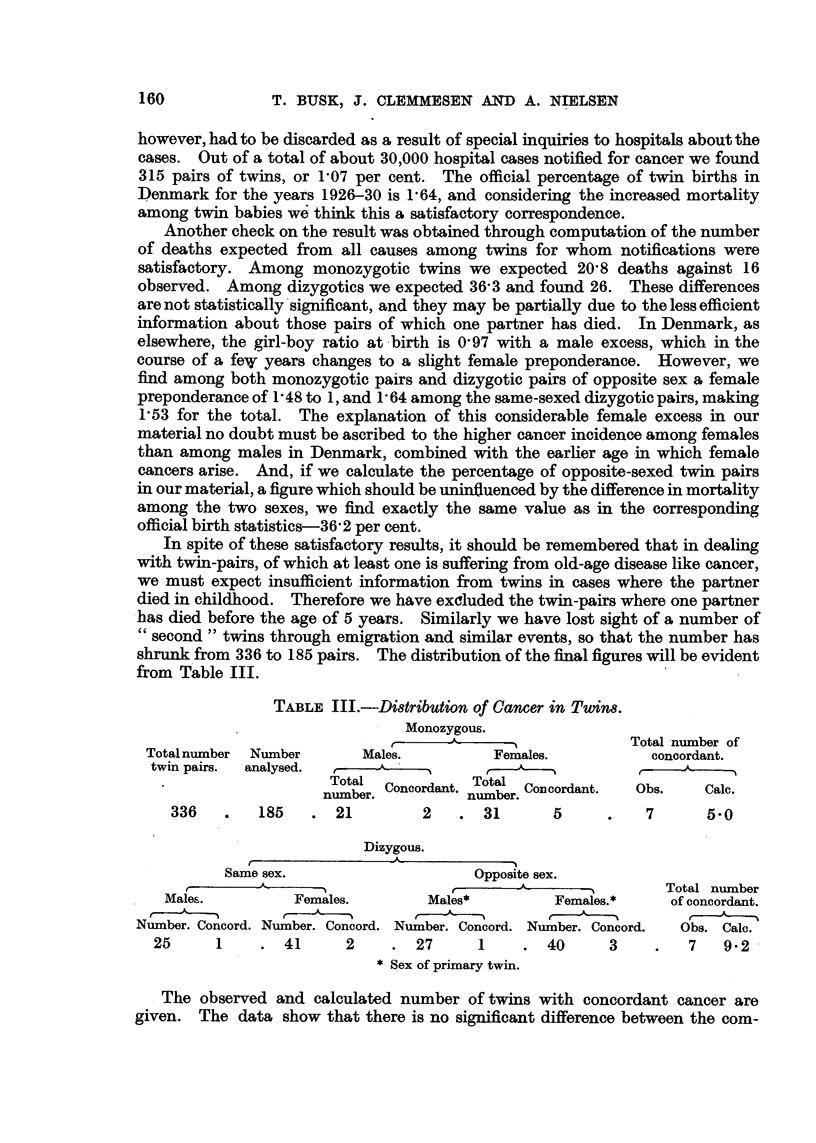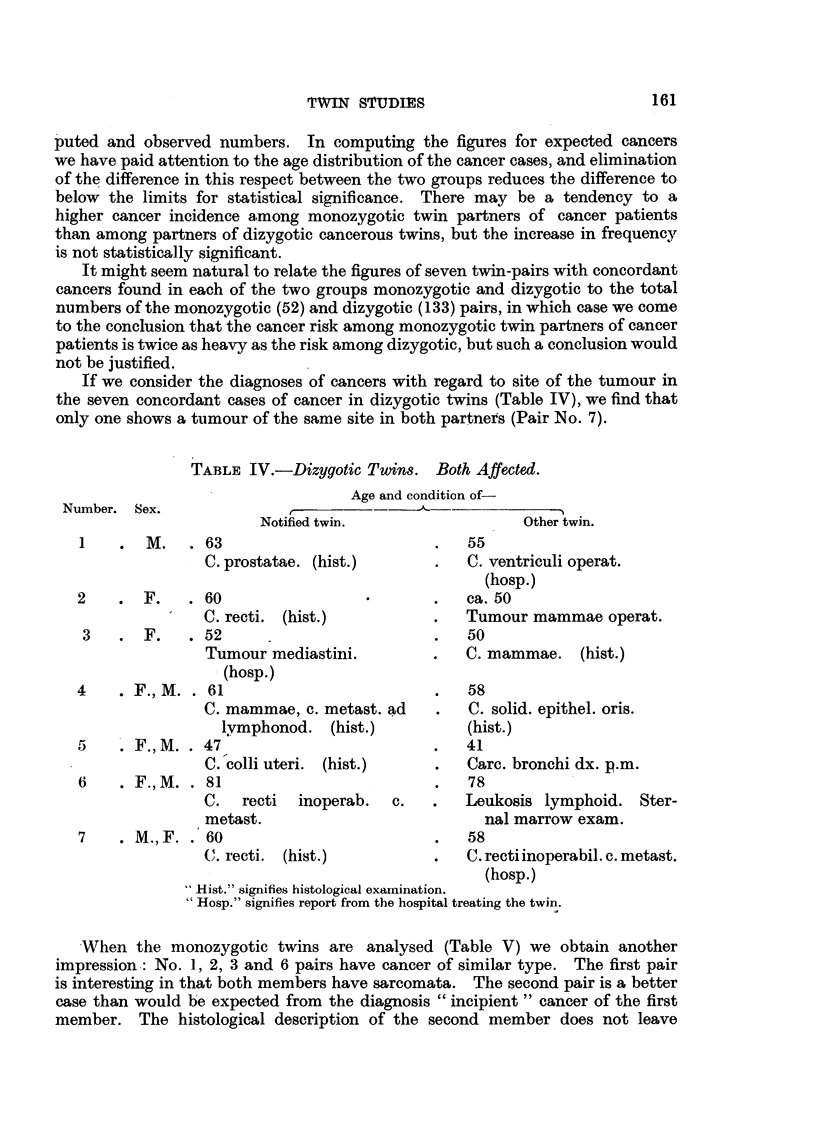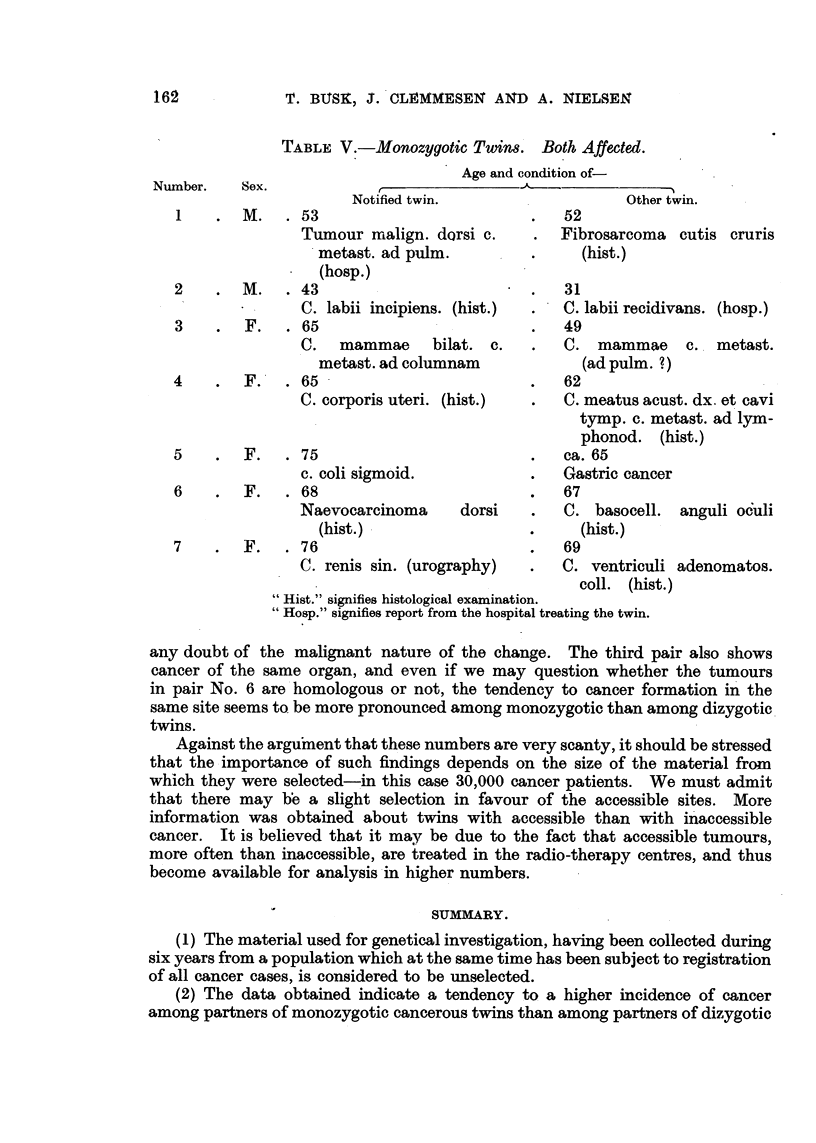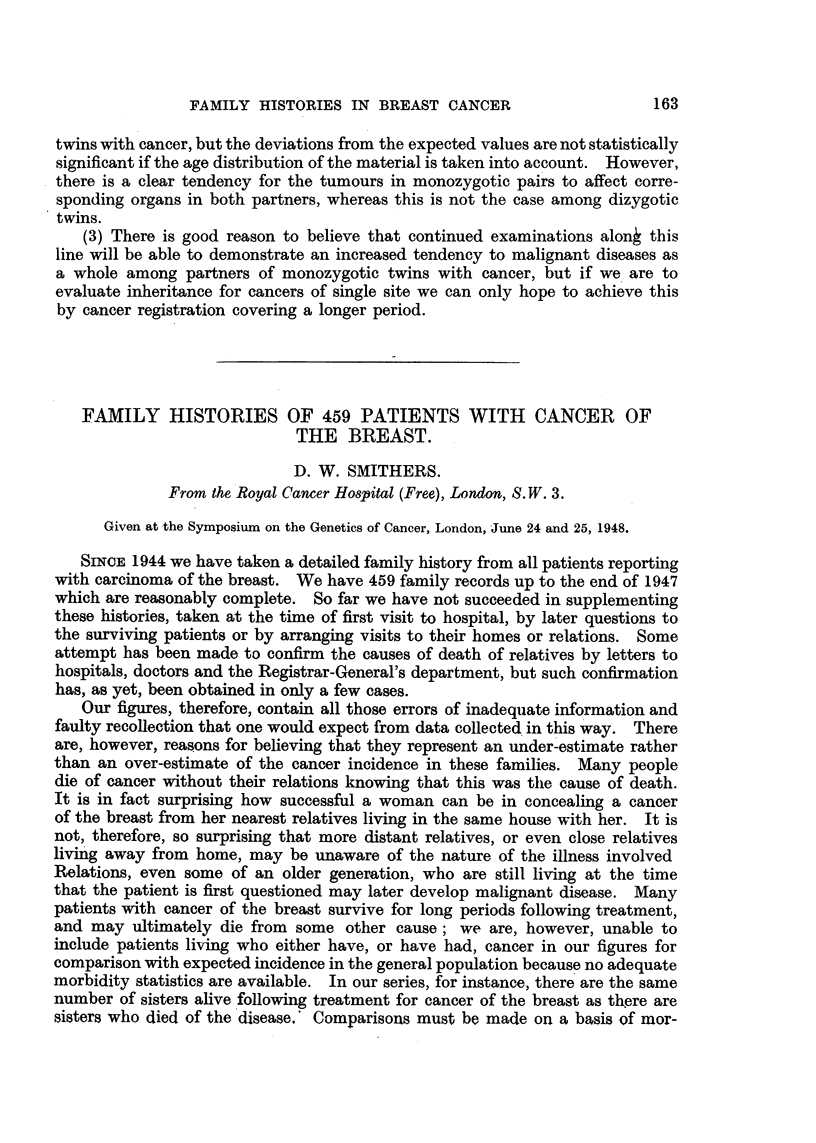# Twin Studies and Other Genetical Investigations in the Danish Cancer Registry

**DOI:** 10.1038/bjc.1948.23

**Published:** 1948-06

**Authors:** T. Busk, J. Clemmesen, A. Nielsen


### TWIN STUDIES AND OTHER GENETICAL INVESTIGATIONS IN THE DANISH CANCER REGISTRY.

T. BUSK, J. CLEMMESEN AND A. NIELSEN.

*From the Danish Cancer Registry under The National Anti-Cancer League, Copenhagen.*

Given at the Symposium on the Genetics of Cancer, London, June 24 and 25, 1948.

THE Danish Cancer Registry, founded in 1942 by the National Anti-Cancer League, and working in close collaboration with the National Health Service, is an organization with the primary intention to register all cancer cases, for research purposes. This is done through a voluntary system ; all hospitals notify their cancer cases to the registry, and these notifications are supplemented with death certificates for all persons who are known to have suffered from malignant diseases, including leukaemias, myelomata and brain tumours.

From Clemmesen's studies on occupational mortality from cancer in Denmark it was clear, already before the start of the Registry, that a complete investigation of the occurrence of cancer demanded at least an estimation of the role played by heredity in the origin of various cancers. It was thought to be especially valuable to carry out such an investigation alongside the mapping out of cancer incidence with regard to age, occupation, and other variables, and to compare the hereditary tendency shown by cancers of various sites in the same population and at the same time.

So collaboration was established with Professor Kemp's Institute for Human Genetics supported by mutual grants. Dr. Jacobsen (surgeon) investigated the heredity of breast cancer, and Dr. Videbaek (physician) studied leukaemia, in collaboration with the Institute for Pathological Anatomy. Dr. Brøbech (radiologist) compared the heredity of cervical and body cancers of the uterus, and Dr. Feilberg (surgeon) is analysing the genetical basis of multiple cancers. The financial support is given by grants from King Christian X Fund.

We believe that the results so far obtained have been more encouraging from an oncological than from a genetical point of view. While the methods employed have made it possible to show a practically demonstrable hereditary tendency to breast cancer, followed by a tendency to cancers of all sites, and while leukaemia and cancers of all sites show similar features, the mode of inheritance is difficult to work out, because of the less reliable information obtainable from relatives more remote than sisters, brothers or parents of the patients.

An efficient examination of the mode of inheritance could no doubt be carried out by means of a registration system for cancer patients covering a few generations. So far our registration system has not been made complete, but in order to exploit fully the opportunities at hand we have examined—as a link in the system of genetical investigations—the occurrence of cancer in twins among the cancer cases notified from Danish hospitals in the first six years of the registry, 1942–1947, realising that an unselected sample of such cases would be of some interest.

HEREDITY IN BREAST CANCER AND LEUKAEMIA.

Jacobsen, in his monograph *Heredity in Breast Cancer*, examined the occurrence of cancer in the familes of 200 breast cancer cases from the files of the Cancer Registry, and compared them with 200 control families (Table I).

TABLE I.—*Jacobsen's Breast Cancer and Control Propositae. Observed and Computed Number of Cancer Cases among Relatives.*

(200 *breast cancer cases.*)

| Relatives. | Breast cancer propositae. Number of cases of cancer. | | | | Control propositae. Number of cases of cancer. | | | |
|---|---|---|---|---|---|---|---|---|
| | All sites. | | Breast cancer. | | All sites. | | Breast cancer | |
| | Obs. | Comp. | Obs. | Comp. | Obs. | Comp. | Obs. | Comp. |
| Fathers | 40 | 23 | | | 19 | 22 | | |
| Brothers | 16 | 8 | | | 4 | 10 | | |
| Maternal grand-fathers | 17 | 22 | | | 6 | 20 | | |
| Paternal grand-fathers | 13 | 20 | | | 5 | 19 | | |
| Maternal uncles | 24 | 23 | | | 3 | 21 | | |
| Paternal uncles | 15 | 25 | | | 1 | 31 | | |
| Male relatives | 125 | 121 | | | 38 | 123 | | |
| Mothers | 55 | 29 | 21 | 7 | 19 | 27 | 2 | 4 |
| Sisters | 30 | 16 | 13 | 5 | 8 | 12 | 2 | 2 |
| Maternal grand-mothers | 23 | 33 | 4 | 7 | 4 | 29 | | 4 |
| Paternal grand-mothers | 11 | 26 | 4 | 6 | 3 | 27 | 2 | 4 |
| Maternal aunts | 51 | 43 | 17 | 11 | 6 | 34 | | 5 |
| Paternal aunts | 30 | 30 | 12 | 7 | 3 | 26 | 2 | 4 |
| Female relatives | 200 | 177 | 71 | 43 | 43 | 155 | 8 | 23 |

Nearly all cancer cases among the relatives were checked by means of case-records from hospitals or death certificates. This applies, without exception to the nearer relatives, and both to test and control material. As there was some doubt as to the absolute validity of this control method, we computed the number of cancer cases which would be expected, based on the morbidity figures from the Cancer Registry and on the official mortality statistics. The data of Table I show that the value of Jacobsen's control material is rather doubtful with regard to the groups of more remote relatives. For the groups of closer relatives, as parents and sibs, however, it is fully justified to draw conclusions with regard both to breast cancer and to cancer of all sites, both of which show a definite excess for relatives of breast cancer cases.

Videbaek, working on cases with leukaemia (Table II), made a careful collection of control material. It is clear from our analysis that he succeeded to some extent only ; but the data show that there is still a significant excess of cancer among fathers and sisters of leukaemia patients.

TABLE II.—*Videbaek's Leukaemia and Control Propositi. Observed and Computed Number of Cancer Deaths among Relatives.*

(209 *leukaemia cases.*)

| Relatives. | Leukaemia propositi. | | | Control propositi. | | |
|---|---|---|---|---|---|---|
| | Obs. | Comp. I. | Comp. II. | Obs. | Comp. | Obs./comp. per cent. |
| Fathers | 31 | 20 | | 18 | 19 | 95 |
| Brothers | 18 | 15 | | 9 | 14 | 66 |
| Maternal grandfathers | 21 | 21 | | 17 | 21 | 79 |
| Paternal grandfathers | 22 | 22 | 14 | 13 | 26 | 50 |
| Maternal uncles | 31 | 28 | 17 | 20 | 29 | 68 |
| Paternal uncles | 26 | 30 | 19 | 18 | 30 | 59 |
| Sons | | 1 | | 1 | 1 | |
| Male relatives | 149 | 137 | | 96 | 140 | 68 |
| Mothers | 28 | 24 | | 21 | 22 | 95 |
| Sisters | 32 | 18 | | 15 | 17 | 87 |
| Maternal grand-mothers | 23 | 27 | | 27 | 29 | 92 |
| Paternal grand-mothers | 22 | 28 | 17 | 16 | 23 | 69 |
| Maternal aunts | 33 | 40 | 24 | 22 | 36 | 61 |
| Paternal aunts | 31 | 40 | 25 | 20 | 32 | 62 |
| Daughters | 1 | 1 | | 1 | 1 | |
| Female relatives | 170 | 178 | | 122 | 160 | 76 |
| All relatives | 319 | 315 | | 218 | 300 | 73 |

These investigations into the heredity of breast cancer and leukaemia illustrate that even under very favourable conditions which prevail in a small country, and with easy access to families interested in such investigations, it is very difficult indeed to obtain a satisfactory control material. Nevertheless, the data so far obtained show that there is a definite hereditary tendency to breast cancer, combined with a tendency to cancers of all sites, and a corresponding phenomenon was found with regard to leukaemia and cancers of all sites.

It has been reported by various authors that in families with a hereditary predisposition for cancer, the disease will appear at an earlier age in the younger generations. Thus Videbaek found an " anticipation " in material gathered from the literature, but it can be demonstrated on his own material as well as on

Jacobsen's that a higher age of the propositus was followed by a loss in the number of known grandparents, which indicates reduced reliability of even family information from older propositi. Moreover, the younger generation will show a higher number of diagnosed cancers and leukaemias as a consequence of the development of diagnostic facilities. The difference in age at onset of the disease in different generations may, therefore, be due to the method of observation itself, as is shown by the fact that leukaemia and cancer cases observed at about the same time but in different generations will exhibit age differences.

Jacobsen observed that propositae among whose relatives he found cases of cancer were younger than propositae with families without cancer, and he assumed that this was due to hereditary factors. Another explanation, however, seems to be more obvious, viz. that the old propositae are unable to give detailed information about their relatives in earlier generations because these relatives most often died years ago. This assumption is supported by the fact that most of the old propositae are grouped by Jacobsen amongst those with " no hereditary predisposition."

We find it very doubtful whether anticipation and an earlier occurrence of hereditarily predisposed cancer cases can be demonstrated by means of materials collected as those published by Jacobsen and Videbaek. Material collected in a registry does not seem to have the same shortcomings, but a further and presumably final study of such phenomena would, however, demand a cancer registration through a longer period, and at present this has not been achieved anywhere. Yet, our material seems to offer a good opportunity for studies on cancer in twins (and already from the start of the genetical team-work such an investigation had been prepared).

### HEREDITY OF CANCER IN TWINS.

It seems particularly advantageous that we have at our disposal an unselected material which is rather unusual for twin studies of this kind. It comprised originally all hospital cancer cases from the Danish population of 4 million through six years, or an annual number between 5000 and 6000 cases, but this figure must be reduced by about 20 per cent in which the question about twin birth had been left unanswered, giving a total very near to 30,000 cases (29,458).

The system of notification involves a rather heavy toll of omissions, yet it has functioned satisfactorily. The special questions asked twins with cancer—besides such items as name, address, occupation and the like—were confined to previous diseases of a more serious character, including diagnosis and the date, and place of treatment. In order to identify whether the twins were monozygotic or dizygotic, questions were asked about the similarity and about mistakes made by parents or others, and similarly about type and colour of hair, colour of eyes, stature, height and weight. On the whole it may be said that we demanded almost full congruity before accepting a pair as monozygotic. The final distribution of dizygotic twins on groups of same and opposite sex, and the proportion between monozygotic and dizygotic pairs seem to show that our measures have been justified. It should be stressed that we have not been in a position to contact the patients or their twins personally. We have had to rely on information from the hospital doctors, and, on the whole, our confidence in them has been justified. A number of cases in which both twins were affected, however, had to be discarded as a result of special inquiries to hospitals about the cases. Out of a total of about 30,000 hospital cases notified for cancer we found 315 pairs of twins, or 1·07 per cent. The official percentage of twin births in Denmark for the years 1926–30 is 1·64, and considering the increased mortality among twin babies we think this a satisfactory correspondence.

Another check on the result was obtained through computation of the number of deaths expected from all causes among twins for whom notifications were satisfactory. Among monozygotic twins we expected 20·8 deaths against 16 observed. Among dizygotics we expected 36·3 and found 26. These differences are not statistically significant, and they may be partially due to the less efficient information about those pairs of which one partner has died. In Denmark, as elsewhere, the girl-boy ratio at birth is 0·97 with a male excess, which in the course of a few years changes to a slight female preponderance. However, we find among both monozygotic pairs and dizygotic pairs of opposite sex a female preponderance of 1·48 to 1, and 1·64 among the same-sexed dizygotic pairs, making 1·53 for the total. The explanation of this considerable female excess in our material no doubt must be ascribed to the higher cancer incidence among females than among males in Denmark, combined with the earlier age in which female cancers arise. And, if we calculate the percentage of opposite-sexed twin pairs in our material, a figure which should be uninfluenced by the difference in mortality among the two sexes, we find exactly the same value as in the corresponding official birth statistics—36·2 per cent.

In spite of these satisfactory results, it should be remembered that in dealing with twin-pairs, of which at least one is suffering from old-age disease like cancer, we must expect insufficient information from twins in cases where the partner died in childhood. Therefore we have excluded the twin-pairs where one partner has died before the age of 5 years. Similarly we have lost sight of a number of "second" twins through emigration and similar events, so that the number has shrunk from 336 to 185 pairs. The distribution of the final figures will be evident from Table III.

TABLE III.—*Distribution of Cancer in Twins.*

| Total number twin pairs. | Number analysed. | Monozygous. Males. Total number. | Monozygous. Males. Concordant. | Monozygous. Females. Total number. | Monozygous. Females. Concordant. | Total number of concordant. Obs. | Total number of concordant. Calc. |
|---|---|---|---|---|---|---|---|
| 336 | 185 | 21 | 2 | 31 | 5 | 7 | 5·0 |

| Dizygous. Same sex. Males. Number. | Concord. | Same sex. Females. Number. | Concord. | Opposite sex. Males* Number. | Concord. | Opposite sex. Females.* Number. | Concord. | Total number of concordant. Obs. | Calc. |
|---|---|---|---|---|---|---|---|---|---|
| 25 | 1 | 41 | 2 | 27 | 1 | 40 | 3 | 7 | 9·2 |

* Sex of primary twin.

The observed and calculated number of twins with concordant cancer are given. The data show that there is no significant difference between the computed and observed numbers. In computing the figures for expected cancers we have paid attention to the age distribution of the cancer cases, and elimination of the difference in this respect between the two groups reduces the difference to below the limits for statistical significance. There may be a tendency to a higher cancer incidence among monozygotic twin partners of cancer patients than among partners of dizygotic cancerous twins, but the increase in frequency is not statistically significant.

It might seem natural to relate the figures of seven twin-pairs with concordant cancers found in each of the two groups monozygotic and dizygotic to the total numbers of the monozygotic (52) and dizygotic (133) pairs, in which case we come to the conclusion that the cancer risk among monozygotic twin partners of cancer patients is twice as heavy as the risk among dizygotic, but such a conclusion would not be justified.

If we consider the diagnoses of cancers with regard to site of the tumour in the seven concordant cases of cancer in dizygotic twins (Table IV), we find that only one shows a tumour of the same site in both partners (Pair No. 7).

TABLE IV.—*Dizygotic Twins. Both Affected.*

| Number. | Sex. | Age and condition of— Notified twin. | Age and condition of— Other twin. |
|---|---|---|---|
| 1 | M. | 63<br>C. prostatae. (hist.) | 55<br>C. ventriculi operat. (hosp.) |
| 2 | F. | 60<br>C. recti. (hist.) | ca. 50<br>Tumour mammae operat. |
| 3 | F. | 52<br>Tumour mediastini. (hosp.) | 50<br>C. mammae. (hist.) |
| 4 | F., M. | 61<br>C. mammae, c. metast. ad lymphonod. (hist.) | 58<br>C. solid. epithel. oris. (hist.) |
| 5 | F., M. | 47<br>C. colli uteri. (hist.) | 41<br>Carc. bronchi dx. p.m. |
| 6 | F., M. | 81<br>C. recti inoperab. c. metast. | 78<br>Leukosis lymphoid. Sternal marrow exam. |
| 7 | M., F. | 60<br>C. recti. (hist.) | 58<br>C. recti inoperabil. c. metast. (hosp.) |

" Hist." signifies histological examination.
" Hosp." signifies report from the hospital treating the twin.

When the monozygotic twins are analysed (Table V) we obtain another impression : No. 1, 2, 3 and 6 pairs have cancer of similar type. The first pair is interesting in that both members have sarcomata. The second pair is a better case than would be expected from the diagnosis " incipient " cancer of the first member. The histological description of the second member does not leave

TABLE V.—*Monozygotic Twins. Both Affected.*

| Number. | Sex. | Age and condition of— Notified twin. | Other twin. |
|---|---|---|---|
| 1 | M. | 53 Tumour malign. dorsi c. metast. ad pulm. (hosp.) | 52 Fibrosarcoma cutis cruris (hist.) |
| 2 | M. | 43 C. labii incipiens. (hist.) | 31 C. labii recidivans. (hosp.) |
| 3 | F. | 65 C. mammae bilat. c. metast. ad columnam | 49 C. mammae c. metast. (ad pulm. ?) |
| 4 | F. | 65 C. corporis uteri. (hist.) | 62 C. meatus acust. dx. et cavi tymp. c. metast. ad lymphonod. (hist.) |
| 5 | F. | 75 c. coli sigmoid. | ca. 65 Gastric cancer |
| 6 | F. | 68 Naevocarcinoma dorsi (hist.) | 67 C. basocell. anguli oculi (hist.) |
| 7 | F. | 76 C. renis sin. (urography) | 69 C. ventriculi adenomatos. coll. (hist.) |

" Hist." signifies histological examination.
" Hosp." signifies report from the hospital treating the twin.

any doubt of the malignant nature of the change. The third pair also shows cancer of the same organ, and even if we may question whether the tumours in pair No. 6 are homologous or not, the tendency to cancer formation in the same site seems to be more pronounced among monozygotic than among dizygotic twins.

Against the argument that these numbers are very scanty, it should be stressed that the importance of such findings depends on the size of the material from which they were selected—in this case 30,000 cancer patients. We must admit that there may be a slight selection in favour of the accessible sites. More information was obtained about twins with accessible than with inaccessible cancer. It is believed that it may be due to the fact that accessible tumours, more often than inaccessible, are treated in the radio-therapy centres, and thus become available for analysis in higher numbers.

### SUMMARY.

(1) The material used for genetical investigation, having been collected during six years from a population which at the same time has been subject to registration of all cancer cases, is considered to be unselected.

(2) The data obtained indicate a tendency to a higher incidence of cancer among partners of monozygotic cancerous twins than among partners of dizygotic twins with cancer, but the deviations from the expected values are not statistically significant if the age distribution of the material is taken into account. However, there is a clear tendency for the tumours in monozygotic pairs to affect corresponding organs in both partners, whereas this is not the case among dizygotic twins.

(3) There is good reason to believe that continued examinations along this line will be able to demonstrate an increased tendency to malignant diseases as a whole among partners of monozygotic twins with cancer, but if we are to evaluate inheritance for cancers of single site we can only hope to achieve this by cancer registration covering a longer period.